# Determination of Ten Flavonoids in the Raw and Fermented Fructus Aurantii by Quantitative Analysis of Multicomponents via a Single Marker (QAMS) Based on UPLC

**DOI:** 10.1155/2023/6067647

**Published:** 2023-06-03

**Authors:** Ting Yang, Yingying Huang, Qinru Li, Qijian Xu, Yangbing Fang, Jiangling Long, Aihua Huang, Meiqi Wang, Quan Xia

**Affiliations:** School of Pharmaceutical Sciences, Guangzhou University of Chinese Medicine, Guangzhou 510006, Guangdong, China

## Abstract

Fermented Fructus Aurantii (FFA) is widely used in South China for the treatment of functional dyspepsia. Naringin, neohesperidin, and other flavonoids are the main pharmacodynamic components of FFA. A new method is presented for the simultaneous determination of 10 flavonoids (including flavonoid glycosides and aglycones) in FFA using the quantitative analysis of multicomponents via a single marker (QAMS) approach and is used to investigate changes in flavonoids during fermentation. The viability and precision of QAMS were validated against the ultrahigh-performance liquid chromatography (UPLC), with various UPLC instruments and chromatographic conditions being evaluated. Differences between raw Fructus Aurantii (RFA) and FFA were examined using orthogonal partial least squares discrimination analysis (OPLS-DA) and content determination. The influence of various fermentation conditions on flavonoids was also investigated. There were no appreciable differences between the QAMS and the external standard method (ESM), demonstrating that QAMS is an improved method for the determination of FA and FFA. FFA and RFA can be readily distinguished based on OPLS-DA chemometric modelling and the corresponding chromatograms. In addition, the flavonoid changes after fermentation. Fermentation considerably reduced the contents of flavonoid glycosides, while increasing hesperidin-7-O-glucoside and flavonoid aglycones. Moreover, fermentation conditions impact multiple flavonoids in FA, so controlling these conditions is necessary for the quality control of fermented FA products. This QAMS approach is useful for detecting numerous components in RFA and FFA simply, quickly, and efficiently, thus strengthening the quality control of FA and its fermented products.

## 1. Introduction

Fructus Aurantiic (FA), also known as *Zhiqiao* in China, is derived from the fruit of the *Citrus aurantium* L. plant and its cultivars [1]. It is a famous and popularly used herbal medicine that is widely used around the world, particularly in China, Japan, India, and Vietnam. FA is often used for the clinical treatment of stomach distension, gastrointestinal food retention, and uterine prolapse [2, 3]. Many studies have shown that FA contains various active ingredients, with flavonoids being the most active [4, 5]. Naringin and neohesperidin are the significant flavonoids in FA and were selected for quantitative analysis in the quality control of FA for the 2020 edition of *The Chinese Pharmacopoeia* [1]. In South China (e.g., Guangdong, Hong Kong, and Macao), fermented Fructus Aurantii (FFA) is more widely used because of its improved efficacy in the treatment of functional dyspepsia [6]. Whilst our previous research found that flavonoids are also the main active components in FFA, the contents of naringin and neohesperidin were lower, indicating that other flavonoids may have pharmacodynamic roles. A comprehensive analysis of the flavonoid content will be valuable in the evaluation of FFA quality.

The curative effects of traditional Chinese medicines (TCM) are related to their complex chemical components [7]. The chemical composition of FA is more complex following fermentation, so quantitative examination of only two flavonoids is insufficient to comprehensively evaluate its quality. While multicomponent quality control methods are desirable for Chinese herbal medicines with multiple targets, they can be problematic. However, the quantitative analysis of multicomponents via a single marker (QAMS) method can be useful [8]. QAMS is an analytical method that can simultaneously monitor multiple analytes via the determination of a cheap and readily available reference compound. This widely used approach significantly alleviates the deficiency and high cost of reference compounds [9].

**Figure 1:**
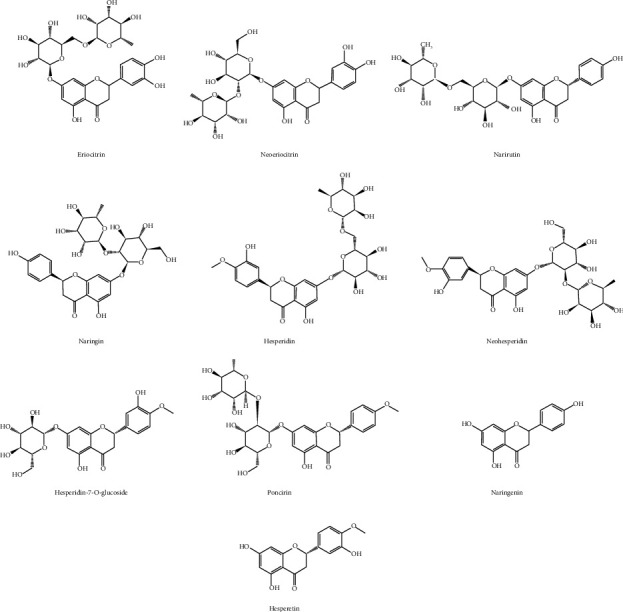
Chemical structure of ten flavonoids.

This study investigated the viability and applicability of the QAMS method. Ten flavonoids that exhibit apparent changes after fermentation were selected and accurately quantified using ultrahigh-performance liquid chromatography (UPLC). Changes in these compounds due to fermentation were compared using the external standard method (ESM) and the newly developed QAMS method. The QAMS approach can shorten the detection time to 30 min, improve efficiency, and reduce analytical costs [10–15]. Moreover, fermentation conditions affect microbial metabolism during the fermentation of FA and, consequently, alter the contents of various chemical components. This study used the QAMS method to simultaneously determine the contents of the ten flavonoids in FA and FFA (Figure 1) under different fermentation conditions, namely, fermentation time, temperature, humidity, and soaking time and provide a foundation for the optimization of FFA production. This study also includes a preliminary exploration of the changes in flavonoids during the fermentation of FA and a scientific basis for the quality control and evaluation of FA and its processed products (FFA).

## 2. Materials and Methods

### 2.1. Materials and Chemicals

The Fructus Aurantii samples were procured from the Guangzhou Zhixin Group and authenticated by Prof. Danyan Zhang from the Department of Resources and Identification of Chinese Herbal Medicine at Guangzhou University of Traditional Chinese Medicine. After determination, the contents of naringin and neohesperidin in FA were no less than 4% and 3%, respectively, meeting the requirements of *the Chinese Pharmacopoeia* [1]. Phosphoric acid of chromatographic quality was acquired from the Guangzhou Chemical Reagent Co., Ltd. (Guangzhou, China). The experiment employed ultrapure water. Shanghai Yuanye Co., Ltd. provided the following compounds: naringin (no: YJ77D9F001), hesperidin (no: P06D9F77001), neohesperidin (no: C05F4Y2), naringenin (no: YJ0603HA13), and hesperetin (no: C03F6Y1). The following compounds were bought from Chengdu Pu Si Biotechnology Co., Ltd.: narirutin (no: PS011543), eriocitrin (no: PS010198), neoeriocitrin (no: PS010420), hesperidin-7-O-glucoside (no: PS020721), and poncirin (no: PS010580). The purity of 10 standards exceeded 98% (the structure of the ten flavonoids are shown in Figure 1) (the picture of FFA and FA are shown in Supporting Information Figure S1).

### 2.2. Instruments and Conditions

Two UPLC systems were used in this research: a Shimadzu LC-20A series UPLC system (Shimadzu, Japan) and a Waters Acquity UPLC system (Waters, USA). Both systems had an autosampler, an online degasser, a photodiode array detector, binary pumps, and a column heater. For sample separation, the following three analytical columns were utilized: Waters UPLC BEH C_18_ (2.1 mm × 100 mm, 1.6 *μ*m), Phenomenex LC C_18_ (2.1 mm × 100 mm, 1.6 *μ*m), and Shimadzu C_18_ (2.1 mm × 100 mm, 1.6 *μ*m). The mobile phase included the following two components: mobile phase A, which was a 0.1% aqueous solution of phosphoric acid, and aqueous solution B, which contained acetonitrile. The elution conditions followed a solvent gradient, with 5% (B) used for the first 2 min, followed by a gradual increase to 26% (B) over the next 8 min. The concentration of B was increased to 27.7% between 10 and 20 min and then to 55% between 20 and 26 min. Between 26 and 28 minutes, the concentration of B was reduced to 20% and finally to 5% between 28 and 29 min, before being held constant at 5% for the final min. The mobile phase flowed at a rate of 0.3 mL/min throughout the entire detection procedure. A column heater was used to maintain the analysis column at 35°C. The detection wavelength was configured to 283 nm for the UPLC analysis, while the detection volume of the sample was injected into 2 *μ*L.

### 2.3. Processing of FFA Samples

The FFA samples were prepared using the fermentation processing methods outlined in the 1984 edition of the Traditional Chinese Medicine Processing Code of Guangdong Province [6] (the processing steps are shown in Supporting Information Figure S2). The basic operations are as follows: The FA was weighed, with approximately 40 g per batch, and soaked in water for a specified duration of time (2 h, 4 h, 6 h, 8 h, or 10 h). Then, the water was poured out, the soaked FA was placed into a breathable and leaky transparent bag, and the fermentation was performed in a culture incubator with a certain temperature (22°C, 27°C, 32°C, 37°C, or 42°C) and humidity (50%, 60%, 60%, 70%, 80%, 80%, or 90%). After fermentation for a certain period of time (2 d, 3 d, 4 d, 5 d, or 6 d), the FFA samples in the incubator were removed, cleaned, sliced, and dried, and then, the corresponding FFA samples were collected (the sample information is shown in Table 1)

### 2.4. Preparation of Standard and Sample Solutions

To prepare a series of mixed reference results with varying concentrations for UPLC analysis, ten different concentrations of the reference solution were created. Subsequently, different volumes of the reference solution were drawn and mixed before being diluted with methanol. This process resulted in a range of mixed reference solutions with varying concentrations, which were also analyzed using UPLC.

The RFA or FFA samples were dried and then powdered to a particle size of 850 *μ*m. A 0.500 g portion of the powder was extracted by heating under reflux with 45 mL of methanol for 1.5 h. After refluxing and heating, the weight was replenished by adding methanol and shaking the liquid evenly. The resulting supernatant fluid was filtered through a 0.22 *μ*m membrane for UPLC examination.

### 2.5. The Principle of Quantitative Analysis of Multicomponents via a Single Marker Method

The guiding principle of the QAMS method states that the component's content is positively proportional to the detector's response value within a specific linear range [8]. When many components are measured at once, as shown in (1), one of the typical components can be chosen as the internal standard to calculate the relative correction factors (RCF) (*f*_*k/m*_) between the internal and external components. Based on the *f*_*k/m*_ of the components to be evaluated and the internal standard component, an equation can be used to calculate the contents of other components in sample (2) [12].

The calculation of RCF is as follows:

(1) $$\begin{array}{c} f_{k/m}=\frac{f_{k}}{f_{m}}=\frac{\left(W_{k}\times A_{m}\right)}{\left(W_{m}\times A_{k}\right)}\text{.} \end{array}$$

The calculations of the flavonoids' contents are performed as follows:

(2) $$\begin{array}{c} W_{m}=\frac{\left(W_{k}\times A_{M}\right)}{\left(f_{k/m}\times A_{k}\right)}\text{.} \end{array}$$

In this study, *W*_*k*_ and *W*_*m*_ were used to represent the concentrations of naringin and other flavonoids presented in both the FFA samples and reference solutions, while *A*_*k*_ and *A*_*m*_ represent the corresponding peak areas of naringin and these flavonoid components in the abovementioned samples and reference solutions. The values of *f*_*k/m*_ stood for the RCF between naringin and the other nine flavonoids. To assess the effectiveness of QAMS, the contents of 10 flavonoids calculated by QAMS were compared with the results of ESM.

### 2.6. The Quantification of FFA Samples

Each FFA sample was measured three times using both the quantitative analysis of mixture standards (QAMS) and the external standard method (ESM) to determine the contents of 10 flavonoids. To compare the differences in results between the two methods, the standard method difference (SMD) was derived using the following equation, as previously explained [16]:

(3) $$\begin{array}{c} SMD=\left[\frac{\left(W_{QAMS}-W_{ESM}\right)}{W_{QAMS}}\right]\times 100\%\text{.} \end{array}$$

Here, *W*_ESM_ represents the contents of the components measured using the ESM method, while *W*_QAMS_ represents the contents of the components measured using the QAMS method.

### 2.7. Data Processing and Multivariate Statistical Analysis

To classify the samples, the orthogonal partial least squares discrimination analysis (OPLS-DA) method was employed to maximize the covariance between the independent variables *X* and the response variables *Y*. The variable influence on projection (VIP) scores were used to evaluate the discriminatory capacity of each observable variable, with variables possessing a VIP score >1 deemed as potential marker compounds for distinguishing various groupings. All OPLS-DA analyses were conducted by utilizing SIMCA 14.1 software (Umetrics, Umea, Sweden).

## 3. Results and Discussion

### 3.1. Optimization of the Preparation of Sample Solutions

To optimize the extraction of the 10 flavonoids from the FFA samples, various reflux extraction times (60, 90, and 120 min) were evaluated. The lowest extraction efficiency was at 60 min, and there was a minimal difference between 90 and 120 min, but higher extraction efficiency was attained at 90 min (Supporting Information Figure S3).

### 3.2. UPLC Method Validation

Samples for the UPLC method validation were pretreated and analyzed as described above, and linearity, the limit of quantification, and the limit of detection were determined. As shown in Table S1, the regression correlation coefficients of all the 10 flavonoids were above 0.9990, indicating a satisfactory linearity of the calibration curves within the range of contents considered appropriate for quantitative analysis. The 10 flavonoids' average recoveries ranged from 96.07 to 104.32%, whereas RSDs' average recoveries were between 0.02% and 2.74%. The RSD values of 10 flavonoids ranged from 1.03% to 1.22% in the repeatability test. The stability of the sample solutions was verified over 24 hours at 4°C, with results ranging from 1.22% to 2.58% across the time points of 0, 4, 8, 12, 16, 20, and 24 h. These findings indicate that the FFA sample solutions were highly stable for up to 24 hours. Thus, this UPLC method demonstrates acceptable recovery, precision, stability, and repeatability for the reliable determination of all 10 flavonoids (Supporting Information Table S1).

### 3.3. Quantitative Analyses of Ten Flavonoid Components by a Single Marker

#### 3.3.1. Calculation of Relative Correction Factors

The selection of a suitable internal standard is essential for the precise measurement of multiple compounds in TCMs. This study has chosen naringin as an internal standard due to its accessibility, affordability, intermediate retention time, and stability.

RCFs were initially calculated based on the peak area ratio and concentrations of naringin and other flavonoids in the mixed reference standard for the simultaneous determination of the ten flavonoids using QAMS. The RCFs of the nine flavonoids relative to naringin are shown in Table 2. The RCFs displayed excellent accuracy, with RSDs between 0.11% and 2.93%.

#### 3.3.2. Evaluation of the Durability and System Applicability of Quantitative Analysis of Multicomponents by a Single Marker

The impact of varied flow rates, chromatographic columns, and column temperatures on the RCFs was examined to appraise the stability and durability of the QAMS method. Three columns of Waters UPLC BEH C_18_, Phenomenex LC C_18_, and Shimadzu C_18_ columns were used for analysis on a Shimadzu LC-20A UPLC system and a Waters Acquity UPLC system, respectively. The RCFs determined using different instruments and columns exhibited RSDs below 3%. The Shimadzu LC-20A UPLC system equipped with a Waters BEH C_18_ was utilized to assess the influences of column temperature (30, 35, and 40°C) and flow rate (0.1, 0.2, and 0.3 mL/min). The RCFs evaluated using various column temperatures and flow rates had RSDs of less than 3% and 2%, respectively. Thus, instrument, column, column temperature, and flow rate had no significant impact on the RCFs, which also showed good reproducibility (Supporting Information Table S2).

#### 3.3.3. The Location of Target Chromatographic Peaks

The precise determination of target peak locations using a single reference is still a significant problem for QAMS. In order to solve this problem, the concept of relative retention time is proposed to accurately identify the desired chromatographic peak, as outlined in the following equation [17]:

(4) $$\begin{array}{c} t_{m/k}=\frac{t_{m}}{t_{k}}\text{.} \end{array}$$

Here, *t*_*k*_ and *t*_*m*_ are the retention times of naringin and other flavonoids under test, respectively.

Two different UPLC devices were used to evaluate the relative retention times of three chromatographic columns. The findings revealed that their RSD values for the relative retention times of all components were less than 3%, indicating that they could be utilized to locate the peak of all tested components (Supporting Information Table S3).

#### 3.3.4. Consistency Assessment of QAMS and ESM Results

The concentrations of the 10 flavonoids were determined in 20 FFA samples from various regions using both the ESM and QAMS (Table 3). The accuracy of QAMS was expressed as the SMD value by comparing the analytical results. SMD ranged from 0% to 2.8% (Supporting Information Table S4), which demonstrates that it is feasible to simultaneously quantify these 10 flavonoids in FFA samples using QAMS.

### 3.4. Quantitative Analysis of RFA and FFA Samples

#### 3.4.1. Comparison of the Content and Quantity of Flavonoids in RFA and FFA

**Figure 2:**
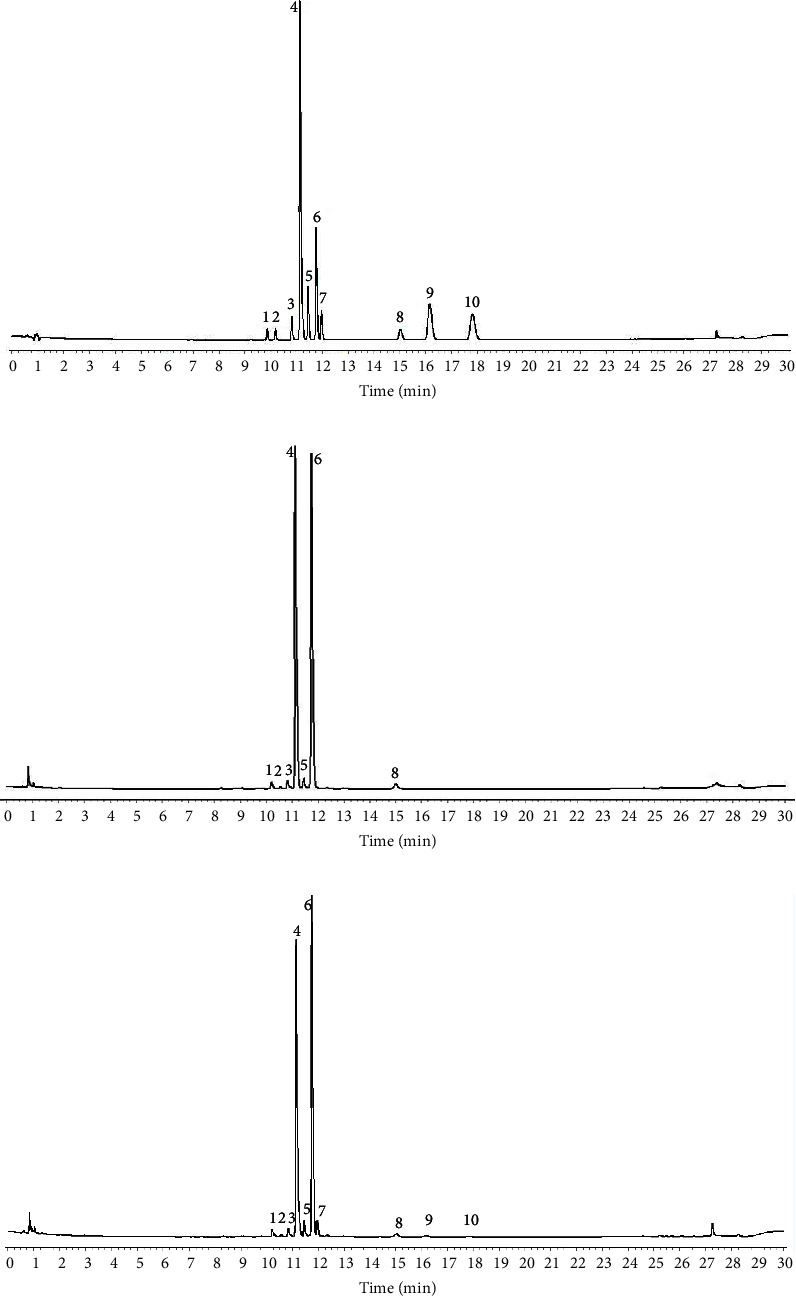
Representative UPLC chromatograms of the standards (a), RFA (b), and FFA samples (c). Peak identification: (1) eriocitrin, (2) neoeriocitrin, (3) narirutin, (4) naringin, (5) hesperidin, (6) neohesperidin, (7) hesperidin-7-O-glucoside, (8) poncirin, (9) naringenin, and (10) hesperetin.

**Figure 3:**
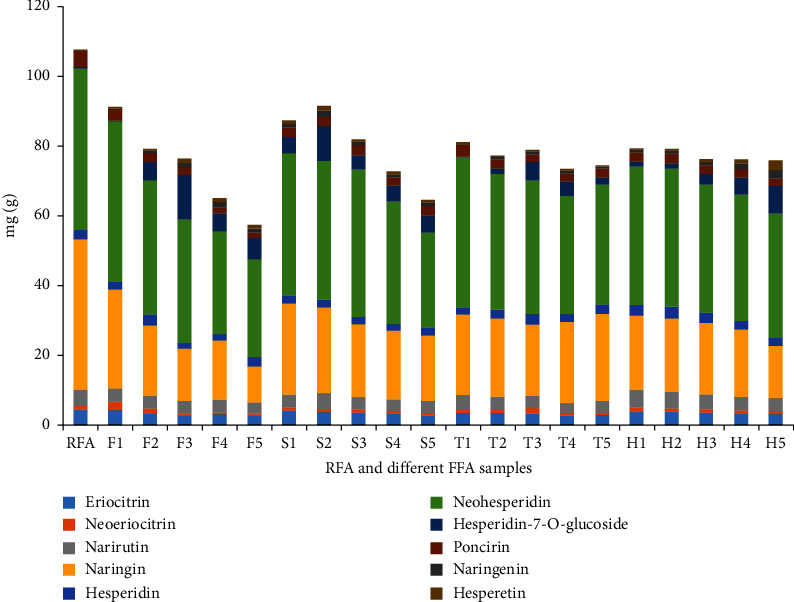
Proportion of 10 components in samples of different fermentation conditions.

Figures 2 and 3 compare the 10 flavonoids in RFA and FFA samples produced under various fermentation conditions. There were seven identifiable peaks in RFA and 10 in the FFA samples, thus revealing three new peaks (Figure 2). The peak heights of flavonoid glycosides were significantly higher in RFA than in FFA, while flavonoid aglycones were distinctly lower in RFA than in FFA.  Figure 3 shows that the contents of the seven flavonoid glycosides were higher in RFA than in all the FFA samples, and RFA contained almost no, or only a few, flavonoid aglycones. Thus, fermentation reduced the flavonoid glycosides and increased the flavonoid aglycones, and it is speculated that the three flavonoid aglycones were produced by the process of fermentation (Supporting Information Table S5–S8).

These data demonstrate the abundant differences in the chemical composition of RFA and FFA. Multivariate statistical analysis (OPLS-DA) was applied to characterize and visualize these differences arising from fermentation.

#### 3.4.2. Multivariate Statistical Analysis of Flavonoids Composition Changes in FA before and after Fermentation (RFA and FFA)

**Figure 4:**
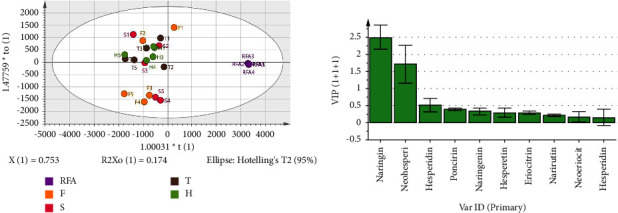
Loading plot obtained by orthogonal partial least square discrimination analysis: score scatter plot (a) and diagram of VIP value (b).

OPLS-DA was used to discriminate between FA samples before and after fermentation based on the flavonoid content. The scores plot in Figure 4(a) shows that the 20 FFA samples were clearly separated from the RFA samples, further illustrating the changes in chemical composition as a result of fermentation. The variable importance plot (VIP) in Figure 4(b) shows the contribution of each flavonoid component to the OPLS-DA model. Naringin and neohesperidin exhibited high VIP values, demonstrating their considerable contribution to sample classification.

Studies have shown that naringin and hesperidin are not easily absorbed from Chinese herbal medicine [18]. However, processing can transform these compounds into single glycosides or aglycones, significantly improving their bioavailability and absorption by the human body [19–21]. Fermentation of FA can produce secondary glycosides such as naringenin-7-O-glucoside and hesperidin-7-O-glucoside, as well as significantly increase the contents of naringenin and hesperetin. It is speculated that some flavonoid glycosides are degraded to aglycones or secondary glycosides by intracellular or extracellular enzymes secreted by microorganisms.

Thus, consistent with the contents analysis of flavonoids, OPLS-DA provides further evidence that fermentation affects the composition of FA, resulting in differences between RFA and FFA. Fermentation conditions impact FFA composition, and further analysis of different conditions is warranted.

### 3.5. Comparison of Ten Flavonoids Components of FFA under Different Fermentation Conditions

**Figure 5:**
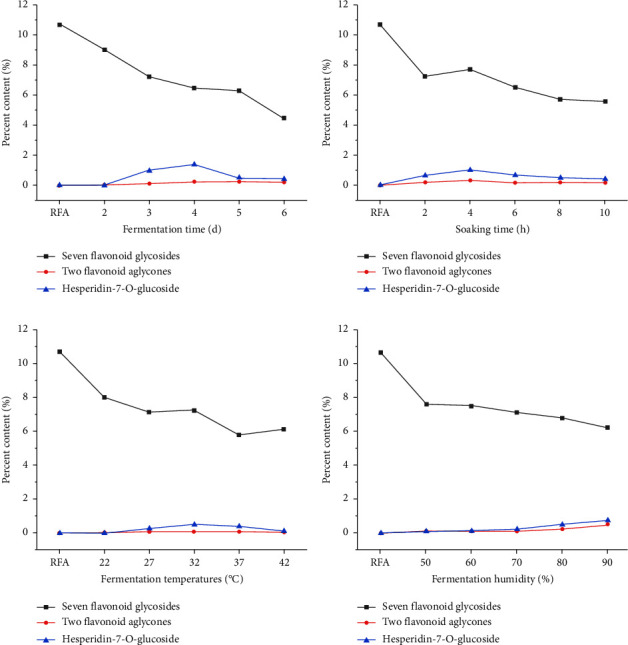
Comparison of the contents of seven flavonoid glycosides, two flavonoid aglycones, and hesperidin-7-(O)-glucoside in RFA and different FFA under different fermentation times (a), different soaking times (b), different fermentation temperatures (c), and different fermentation humidity (d).

Figures 3 and 5 show that fermentation temperature, humidity, time, and soaking time affect the chemical composition of FFA. Flavonoid contents are reduced when the fermentation time reaches four days. Fermentation temperatures over 37°C or below 27°C affect the fermentation process and the production of new compounds. Similarly, when the fermentation humidity is low, the growth of microorganisms is inhibited, thus impacting the fermentation process and the production of new compounds. These trends in the composition of flavonoids in FFA processed under different conditions provide a scientific basis for the optimization of the fermentation process (Supporting Information Table S5∼S8).

## 4. Conclusions

The study of TCMs requires comprehensive analytical methods. The present study establishes a QAMS method for the determination of 10 flavonoids in FFA. This method is shown to be efficient, reliable, and suitable for the evaluation of FFA quality. These 10 flavonoids were determined in FFA and FA to explore the changes arising from the fermentation process. Fermentation conditions (temperature, humidity, and time) affect the flavonoid contents. Fermentation results in a considerable decrease in flavonoid glycosides, while hesperidin-7-O-glucoside and flavonoid aglycones increase. The QAMS method developed in this study will make the quality assessment of FFA more feasible and efficient and will provide a basis for process optimization.

## Figures and Tables

**Table 1:** The names of samples under different fermentation conditions.

Sample	Fermentation time (d)	Soaking time (h)	Fermentation temperatures (°C)	Fermentation humidity (%)
F1	2	4	32	70
F2	3	4	32	70
F3	4	4	32	70
F4	5	4	32	70
F5	6	4	32	70
S1	4	2	32	70
S2	4	4	32	70
S3	4	6	32	70
S4	4	8	32	70
S5	4	10	32	70
T1	4	4	22	70
T2	4	4	27	70
T3	4	4	32	70
T4	4	4	37	70
T5	4	4	42	70
H1	4	4	32	50
H2	4	4	32	60
H3	4	4	32	70
H4	4	4	32	80
H5	4	4	32	90

**Table 2:** RCF values of the ten components of the FFA samples (*n* = 3).

RCF^a^	1 *μ*L	1.5 *μ*L	2 *μ*L	2.5 *μ*L	3 *μ*L	Mean	RSD (%)
*f*_4/1_	0.254	0.252	0.252	0.255	0.256	0.254	0.76
*f*_4/2_	0.327	0.330	0.321	0.343	0.343	0.333	2.93
*f*_4/3_	0.246	0.246	0.241	0.243	0.242	0.243	0.92
*f*_4/4_	1.000	1.000	1.000	1.000	1.000	1.000	0.00
*f*_4/5_	0.532	0.527	0.523	0.522	0.521	0.525	0.85
*f*_4/6_	0.627	0.627	0.628	0.629	0.629	0.628	0.11
*f*_4/7_	0.361	0.356	0.351	0.349	0.348	0.353	1.59
*f*_4/8_	0.267	0.271	0.274	0.275	0.276	0.273	1.29
*f*_4/9_	0.553	0.560	0.565	0.568	0.569	0.563	1.18
*f*_4/10_	0.465	0.479	0.486	0.490	0.493	0.482	2.25

a: 1: eriocitrin; 2: neoeriocitrin; 3: narirutin; 4: naringin; 5: hesperidin; 6: neohesperidin; 7: hesperidin-7-O-glucoside; 8: poncirin; 9: naringenin; 10: hesperetin.

**Table 3:** Contents of the ten components in FFA samples determined by ESM and QAMS methods (mg·g^−1^, *n* = 3).

No.	Nari ngin	Eriocitrin		Neoeriocitrin		Narirutin		Hesperidin		Neohesperidin		Hesperidin-7-O-glucoside		Poncirin		Naringenin		Hesperetin	
		ESM	QAMS	ESM	QAMS	ESM	QAMS	ESM	QAMS	ESM	QAMS	ESM	QAMS	ESM	QAMS	ESM	QAMS	ESM	QAMS
F1	28.42	4.04	4.11	2.62	2.61	3.76	3.76	2.15	2.17	45.16	46.06	0.16	0.16	3.38	3.43	0.17	0.17	0.06	0.06
F2	20.38	3.13	3.14	1.66	1.64	3.46	3.45	3.00	3.05	37.85	38.60	9.66	9.79	2.49	2.50	0.74	0.75	0.52	0.52
F3	17.03	2.94	2.94	0.65	0.64	3.51	3.50	1.87	1.89	27.43	27.49	14.05	14.17	1.58	1.60	1.39	1.37	1.19	1.21
F4	14.94	2.47	2.46	0.87	0.88	3.48	3.47	1.74	1.74	36.76	37.49	5.22	5.26	2.18	2.17	1.28	1.26	1.21	1.23
F5	10.25	2.48	2.46	0.72	0.71	3.38	3.37	2.58	2.60	24.37	24.21	4.37	4.38	1.47	1.45	1.17	1.15	1.02	1.03
S1	18.02	3.82	3.88	0.94	0.95	3.77	3.78	2.31	2.30	40.50	41.11	6.32	6.4	2.99	3.01	0.95	0.96	0.98	0.98
S2	24.08	3.61	3.66	0.94	0.94	4.43	4.47	2.23	2.22	39.00	39.52	10.05	10.18	2.57	2.57	1.78	1.81	1.45	1.46
S3	18.58	3.08	3.09	0.89	0.89	3.39	3.38	2.01	1.98	34.86	35.12	6.67	6.69	2.34	2.32	0.97	0.98	0.80	0.80
S4	18.89	2.49	2.47	0.77	0.75	3.53	3.52	2.23	2.21	27.38	27.18	5.15	5.18	2.45	2.45	1.17	1.18	0.78	0.79
S5	20.93	3.46	3.50	0.90	0.90	3.58	3.57	2.05	2.02	41.63	42.31	4.22	4.22	2.85	2.86	0.92	0.93	0.78	0.79
T1	22.99	3.29	3.32	0.99	1.01	4.18	4.21	2.25	2.24	42.31	43.04	0.16	0.16	3.45	3.48	0.23	0.23	0.17	0.18
T2	22.31	3.21	3.23	1.06	1.08	3.82	3.82	2.68	2.71	35.63	35.94	3.07	3.08	2.49	2.48	0.70	0.71	0.45	0.45
T3	20.38	3.13	3.14	1.68	1.69	3.46	3.45	3.00	3.06	37.85	38.30	5.22	5.26	2.49	2.49	0.74	0.75	0.52	0.52
T4	13.31	2.57	2.55	0.67	0.67	2.99	2.96	2.36	2.36	33.60	33.79	4.19	4.19	2.51	2.51	0.67	0.67	0.50	0.50
T5	15.05	2.70	2.69	0.81	0.80	3.30	3.28	2.72	2.75	34.13	34.34	1.56	1.57	2.56	2.55	0.51	0.51	0.40	0.40
H1	21.29	3.73	3.78	1.02	1.04	5.12	5.19	3.14	3.18	39.10	39.62	1.49	1.49	2.63	2.63	0.73	0.74	0.53	0.53
H2	20.43	3.66	3.71	0.99	1.00	4.73	4.79	3.10	3.14	39.25	39.79	1.71	1.73	2.63	2.64	0.73	0.74	0.68	0.68
H3	20.43	3.44	3.48	0.94	0.94	4.25	4.28	2.70	2.70	36.96	37.35	2.78	2.82	2.62	2.62	0.78	0.79	0.83	0.85
H4	19.23	3.24	3.26	0.91	0.91	3.92	3.93	2.54	2.53	35.77	36.09	5.11	5.15	2.34	2.33	1.64	1.67	1.21	1.19
H5	15.10	2.92	2.93	0.91	0.91	3.70	3.70	2.39	2.36	35.35	35.64	7.75	7.88	2.16	2.14	2.46	2.50	2.87	2.88

## Data Availability

The data used to support the findings of this study are available from the corresponding author upon request.

## Abbreviations

FA: Fructus Aurantii
RFA: Raw Fructus Aurantii
FFA: Fermented Fructus Aurantii
DAD: Diode array detection
ESM: External standard method
QAMS: Quantitative analysis of multicomponents by a single marker
RCF: Relative correction factor
SMD: Standard method difference
TCM: Traditional Chinese medicine
OPLS-DA: Orthogonal partial least squares discrimination analysis.

## Supplementary Materials

Supplementary Materials Supplementary Materials are about “repeatability of the correction factor,” “structure of 10 flavonoids,” and “comparison of components under different fermentation conditions.” Figure S1: Pictures of Fructus Aurantii and fermented Fructus Aurantii. Figure S2: Processing steps of Lingnan Special Decoction Pieces “Processed Fructus aurantia.” Figure S3: Effect of extraction time on extraction yields of the contents of ten components. Table S1: The regression equations, LOD, LOQ, precision, recovery, repeatability, and stability for the determination of ten components. Table S2: Effects of different instruments, columns, column temperatures, and flow rates on RCFs. Table S3: Relative retention time of the nine flavonoids components. Table S4: The standard method difference (SMD) between ESM and QAMS. Table S5–S8: The comparison of the contents of 10 flavonoids in samples under different fermentation conditions.

## References

[1] Chinese Pharmacopoeia Commission (2020). *Pharmacopoeia of People’s Republic of China*.

[2] Zhong G. S. (2016). *Chinese Materia Medica*.

[3] He Y. B., Xu Z. Y. (2013). Xu zhiyings’ experience in using Fructus aurantii. *Zhejiang Journal of traditional Chinese medicine*.

[4] Tan H. (2017). Chemical constituents and pharmacological effects of Fructus aurantii. *Chinese medical guide*.

[5] Li C. X., Yang Y. H., Leng D. S., Liu Y. F. (2019). Research progress on chemical constituents and pharmacological effects of Fructus aurantii. *Journal of Liaoning University of traditional Chinese medicine*.

[6] (1984). *Guangdong Food and Drug Administration Guangdong Provincial Standard for the Processing of Chinese Herbal Pieces*.

[7] Gao H. M., Wang Z. M., Li Y. J., Qian Z. Z. (2011). Overview of the quality standard research of traditional Chinese medicine. *Frontiers of Medicine*.

[8] Zeng S. L., Li S. Z., Lai C. J. S. (2018). Evaluation of anti-lipase activity and bioactive flavonoids in the Citri Reticulatae Pericarpium from different harvest time. *Phytomedicine*.

[9] Li D. W., Zhu M., Shao Y. D., Shen Z., Weng C. C., Yan W. D. (2016). Determination and quality evaluation of green tea extracts through qualitative and quantitative analysis of multi-components by single marker (QAMS). *Food Chemistry*.

[10] Li P., Zeng S. L., Duan L. (2016). Comparison of Aurantii Fructus Immaturus and Aurantii Fructus based on multiple chromatographic analysis and chemometrics methods. *Journal of Chromatography A*.

[11] Zhang J. Z., Gao W. Y., Liu Z., Zhang Z. D., Liu C. X. (2014). Systematic analysis of main constituents in rat biological samples after oral administration of the methanol extract of fructus aurantii by HPLC-ESI-MS/MS. *Iranian Journal of Pharmaceutical Research*.

[12] Li L. L., Zhang S., Xin Y. F. (2018). Role of Role of Quzhou Fructus Aurantii Extract in Preventing and Treating Acute Lung Injury and Inflammationuzhou fructus aurantii extract in preventing and treating acute lung injury and inflammation. *Scientific Reports*.

[13] Wu M., Zhang H. W., Zhou C., Jia H. M., Ma Z., Zou Z. M. (2015). Identification of the chemical constituents in aqueous extract of Zhi-Qiao and evaluation of its antidepressant effect. *Molecules*.

[14] He Y. J., Li Z. K., Wang W. (2018). Chemical Chemical Profiles and Simultaneous Quantification of Aurantii fructus by Use of HPLC-Q-TOF-MS Combined with GC-MS and HPLC Methodsrofiles and simultaneous quantification of aurantii fructus by use of HPLC-Q-TOF-MS combined with GC-MS and HPLC methods. *Molecules*.

[15] Lei Y. T., Wang Y. Q., Sun Z. C. (2020). Quantitative analysis of multicomponents by single marker combined with HPLC fingerprint qualitative analyses for comprehensive evaluation of Aurantii Fructus. *Journal of Separation Science*.

[16] Dong Y. H., Guo Q., Liu J. J., Ma X. Q. (2018). Simultaneous determination of seven phenylethanoid glycosides in Cistanches Herba by a single marker using a new calculation of relative correction factor. *Journal of Separation Science*.

[17] Peng Y., Dong M. H., Zou J., Liu Z. H. (2018). Analysis of the HPLC Analysis of the HPLC Fingerprint and QAMS for Sanhuang Gypsum Soupingerprint and QAMS for sanhuang gypsum soup. *Journal of Analytical Methods in Chemistry*.

[18] Shi S. Q., Yan H., Chen Y. (2020). Pharmacokinetic study of precisely representative antidepressant, prokinetic, anti-inflammatory and anti-oxidative compounds from Fructus aurantii and Magnolia Bark. *Chemico-Biological Interactions*.

[19] Yuan J. B., Wei F. T., Luo X. Z. (2020). Multi-Multi-Component Comparative Pharmacokinetics in Rats After Oral Administration of <i>Fructus aurantii</i> Extract, Naringin, Neohesperidin, and Naringin-Neohesperidinomponent comparative pharmacokinetics in rats after oral administration of fructus aurantii extract, naringin, neohesperidin, and naringin-neohesperidin. *Frontiers in Pharmacology*.

[20] Zhang J. Z., Gao W. Y., Hu X., Liu Z., Liu C. X. (2012). The influence of compatibility of traditional Chinese medicine on the pharmacokinetic of main components in Fructus Aurantii. *Journal of Ethnopharmacology*.

[21] Zhang X. H., Han L. R., Liu J. (2018). Pharmacokinetic Pharmacokinetic Study of 7 Compounds Following Oral Administration of Fructus Aurantii to Depressive Ratstudy of 7 compounds following oral administration of fructus aurantii to depressive rats. *Frontiers in Pharmacology*.

