# Supplementary material for: Determination of Ten Flavonoids in the Raw and Fermented Fructus Aurantii by Quantitative Analysis of Multicomponents via a Single Marker (QAMS) Based on UPLC
Source: J Anal Methods Chem. 2023 Jun 3;2023:6067647. doi: 10.1155/2023/6067647 (PMC10257543; doi:10.1155/2023/6067647)
Supplement: Supplementary Materials — are about “repeatability of the correction factor,” “structure of 10 flavonoids,” and “comparison of components under different fermentation conditions.” Figure S1: Pictures of Fructus Aurantii and fermented Fructus Aurantii. Figure S2: Processing steps of Lingnan Special Decoction Pieces “Processed Fructus aurantia.” Figure S3: Effect of extraction time on extraction yields of the contents of ten components. Table S1: The regression equations, LOD, LOQ, precision, recovery, repeatability, and stability for the determination of ten components. Table S2: Effects of different instruments, columns, column temperatures, and flow rates on RCFs. Table S3: Relative retention time of the nine flavonoids components. Table S4: The standard method difference (SMD) between ESM and QAMS. Table S5–S8: The comparison of the contents of 10 flavonoids in samples under different fermentation conditions. [file 6067647.f1.doc]

**Determination of ten flavonoids in the raw and fermented Fructus aurantii by Quantitative Analysis of multi-components via Single Marker (QAMS) based on UPLC**

SUPPORTIONG INFORMATION


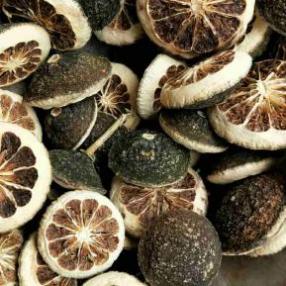

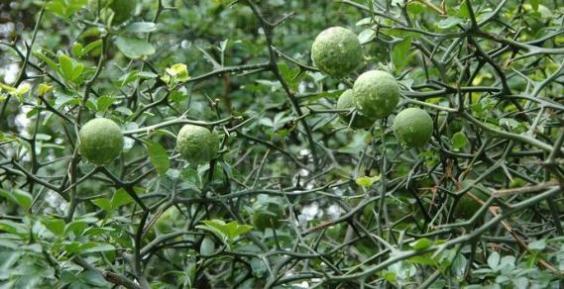

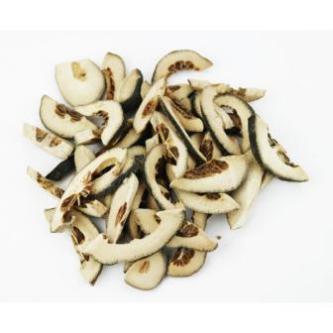

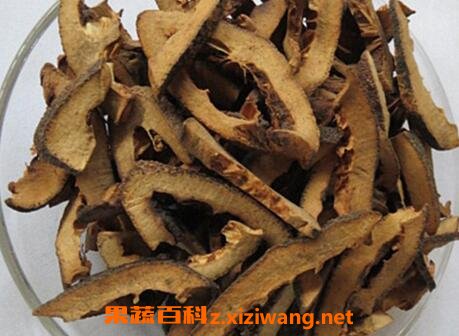


**Fructus aurantii**

**Fermented Fructus aurantii**

**Supplementary Figure S1:** Fructus aurantii and fermented Fructus aurantii


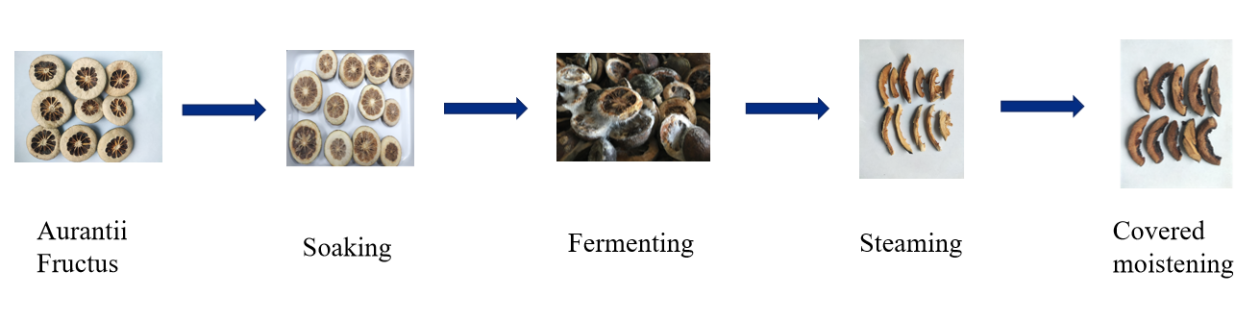

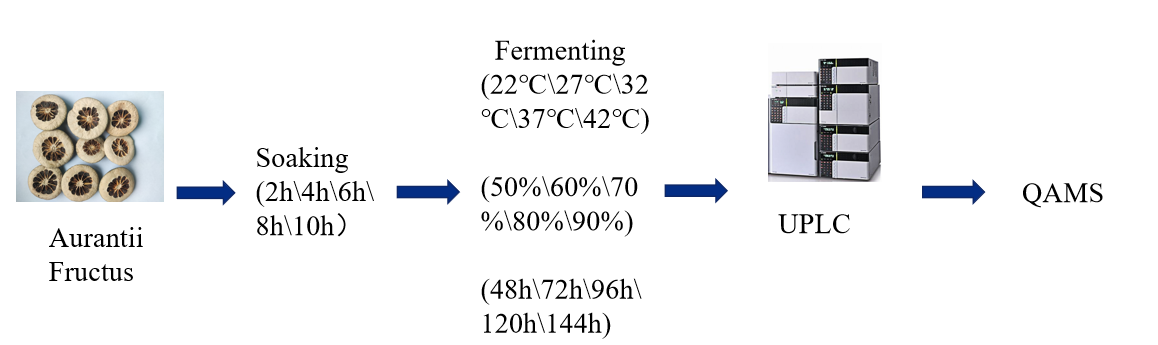


**Supplementary Figure S2:** Processing steps of Lingnan Special Decoction Pieces "Processed Fructus aurantii "


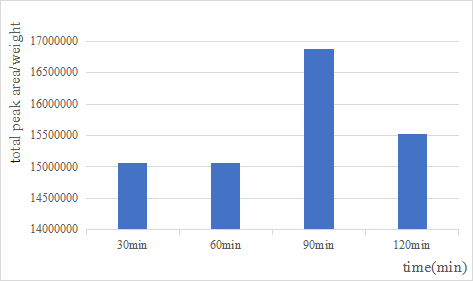


**Supplementary Figure S3:** Effect of extraction time on extraction yields of the contents of ten components

**Supplementary Table S1:** The regression equations, LOD, LOQ, precision, recovery, repeatability and stability for determination of ten components

| **Analytes** | **Regression Equations** | **R^2^** | **Linear Ranges**  **(μg/mL)** | **Precision (%, RSD, n=6)** | |  | **Recovery (n=6)** | |  | **Repeatability**  **(%, RSD, n=6)** | **Stability**  **(%, RSD, n=6)** |  | **LOD/(μg·mL^-1^)** | **LOQ/(μg·mL^-1^)** |
| --- | --- | --- | --- | --- | --- | --- | --- | --- | --- | --- | --- | --- | --- | --- |
|  |  |  |  | **Intra-day** | **Inter-day** |  | **mean** | **RSD (%)** |  |  |  |  |  |  |
| Eriocitrin | y=137686x-10600 | 0.9996 | 0.133～5.3 | 1.12 | 1.22 |  | 101.60 | 1.64 |  | 1.05 | 2.51 |  | 0.12 | 0.40 |
| Neoeriocitrin | y=176065x-8349.3 | 0.9997 | 0.108～4.3 | 2.01 | 2.32 |  | 100.62 | 1.46 |  | 1.21 | 2.58 |  | 0.09 | 0.30 |
| Narirutin | y=110887x-10177 | 0.9996 | 0.343～13.7 | 1.03 | 1.15 |  | 101.01 | 0.06 |  | 1.15 | 1.56 |  | 0.27 | 0.90 |
| Naringin | y=437696x+121634 | 0.9996 | 1.25～50 | 1.21 | 1.33 |  | 103.30 | 0.05 |  | 1.03 | 1.27 |  | 0.51 | 1.69 |
| Hesperidin | y=240959x-25845 | 0.9997 | 0.364～14.55 | 2.01 | 2.23 |  | 101.63 | 1.66 |  | 1.14 | 1.71 |  | 0.28 | 0.93 |
| Neohesperidin | y=339758x-302411 | 0.9996 | 1.25～50 | 1.12 | 1.39 |  | 100.71 | 0.81 |  | 1.08 | 1.22 |  | 0.64 | 2.13 |
| Hesperidin-7-*O*-glucoside | y=161389x-12967 | 0.9997 | 0.335～13.4 | 2.02 | 2.35 |  | 101.65 | 1.71 |  | 1.06 | 1.55 |  | 0.28 | 0.93 |
| Poncirin | y=123841x-6892.8 | 0.9997 | 0.347～13.9 | 1.75 | 1.87 |  | 102.95 | 0.73 |  | 1.10 | 1.47 |  | 0.25 | 0.83 |
| Naringenin | y=245796x-916.81 | 0.9997 | 0.293～11.75 | 1.91 | 1.99 |  | 103.47 | 0.48 |  | 1.09 | 1.59 |  | 0.11 | 0.37 |
| Hesperetin | y=212382x+8594.4 | 0.9998 | 0.316～12.675 | 1.33 | 1.92 |  | 100.19 | 1.55 |  | 1.22 | 1.67 |  | 0.12 | 0.39 |

**Supplementary Table S2:** Effects of different instruments, columns, column temperatures, and flow rates on RCFs (n = 3)

|  |  | Eriocitrin | Neoeriocitrin | Narirutin | Hesp  eridin | Neohesperidin | Hesperidin-7-*O*-glucoside | Poncirin | Naringenin | Hesperetin |
| --- | --- | --- | --- | --- | --- | --- | --- | --- | --- | --- |
| SHIMADZU  LC-20A | Waters BEH C18 | 0.252 | 0.321 | 0.241 | 0.523 | 0.628 | 0.351 | 0.274 | 0.565 | 0.486 |
|  | PHENOMENEX LC C18 | 0.243 | 0.328 | 0.241 | 0.541 | 0.638 | 0.363 | 0.264 | 0.570 | 0.487 |
|  | SHIMADZU C18 | 0.246 | 0.325 | 0.239 | 0.527 | 0.634 | 0.370 | 0.261 | 0.575 | 0.488 |
| Wates Acquity | Waters BEH C18 | 0.243 | 0.313 | 0.237 | 0.538 | 0.633 | 0.348 | 0.268 | 0.568 | 0.472 |
|  | PHENOMENEX LC C18 | 0.245 | 0.315 | 0.238 | 0.538 | 0.635 | 0.351 | 0.277 | 0.566 | 0.479 |
|  | SHIMADZU C18 | 0.244 | 0.313 | 0.245 | 0.534 | 0.630 | 0.350 | 0.274 | 0.563 | 0.472 |
|  | Mean | 0.245 | 0.319 | 0.240 | 0.534 | 0.633 | 0.356 | 0.270 | 0.568 | 0.481 |
|  | RSD  (%) | 1.37 | 2.05 | 1.10 | 1.34 | 0.61 | 2.56 | 2.40 | 0.79 | 1.61 |
| Flow rates  (mL/min) | 0.15 | 0.256 | 0.332 | 0.240 | 0.521 | 0.611 | 0.337 | 0.269 | 0.554 | 0.481 |
|  | 0.2 | 0.258 | 0.337 | 0.242 | 0.526 | 0.632 | 0.351 | 0.261 | 0.573 | 0.501 |
|  | 0.25 | 0.259 | 0.342 | 0.245 | 0.524 | 0.628 | 0.351 | 0.275 | 0.564 | 0.491 |
|  | 0.3 | 0.252 | 0.321 | 0.241 | 0.523 | 0.628 | 0.351 | 0.274 | 0.565 | 0.486 |
|  | 0.35 | 0.252 | 0.328 | 0.247 | 0.523 | 0.631 | 0.356 | 0.263 | 0.570 | 0.512 |
|  | Mean | 0.255 | 0.332 | 0.243 | 0.523 | 0.626 | 0.349 | 0.268 | 0.565 | 0.494 |
|  | RSD  (%) | 1.34 | 2.45 | 1.21 | 0.34 | 1.36 | 2.02 | 2.36 | 1.25 | 2.49 |
| Column  temperatur  es(°C) | 20 | 0.263 | 0.330 | 0.245 | 0.547 | 0.636 | 0.356 | 0.286 | 0.573 | 0.505 |
|  | 25 | 0.266 | 0.340 | 0.247 | 0.541 | 0.636 | 0.360 | 0.282 | 0.583 | 0.515 |
|  | 30 | 0.253 | 0.330 | 0.243 | 0.522 | 0.628 | 0.347 | 0.273 | 0.566 | 0.506 |
|  | 35 | 0.252 | 0.321 | 0.241 | 0.523 | 0.628 | 0.351 | 0.274 | 0.565 | 0.486 |
|  | 40 | 0.258 | 0.327 | 0.247 | 0.528 | 0.631 | 0.361 | 0.284 | 0.583 | 0.507 |
|  | Mean | 0.258 | 0.329 | 0.245 | 0.532 | 0.632 | 0.355 | 0.280 | 0.574 | 0.504 |
|  | RSD  (%) | 2.45 | 2.08 | 1.07 | 2.11 | 0.62 | 1.69 | 2.10 | 1.56 | 2.44 |

**Supplementary Table S3:** Relative retention time of the nine components (n =3 )

| **Instruments** | **columns** | **Eriocitrin** | **Neoeriocitrin** | **Narirutin** | **Hesperidin** | **Neohesperidin** | **Hesperidin-7-O-glucoside** | **Poncirin** | **Naringenin** | **Hesperetin** |
| --- | --- | --- | --- | --- | --- | --- | --- | --- | --- | --- |
| SHIMADZU LC-20A series | Waters BEH C_18_ | 1.239 | 0.435 | 0.276 | 0.380 | 0.808 | 0.983 | 1.896 | 3.684 | 4.249 |
|  | Shimadzu C_18_ | 1.241 | 0.437 | 0.279 | 0.382 | 0.810 | 0.981 | 1.948 | 3.589 | 4.346 |
|  | Phenomenex C_18_ | 1.237 | 0.431 | 0.275 | 0.379 | 0.806 | 0.979 | 1.962 | 3.712 | 4.412 |
| Waters Acquity UPLC system | Waters BEH C_18_ | 1.242 | 0.437 | 0.279 | 0.383 | 0.810 | 0.987 | 1.953 | 3.687 | 4.352 |
|  | Shimadzu C_18_ | 1.243 | 0.438 | 0.281 | 0.385 | 0.804 | 0.99 | 1.955 | 3.690 | 4.354 |
|  | Phenomenex C_18_ | 1.240 | 0.435 | 0.276 | 0.387 | 0.813 | 0.985 | 1.951 | 3.685 | 4.349 |
|  | Mean | 1.24 | 0.44 | 0.28 | 0.38 | 0.81 | 0.98 | 1.94 | 3.67 | 4.34 |
|  | RSD(%) | 0.17 | 0.58 | 0.84 | 0.79 | 0.40 | 0.41 | 1.24 | 1.17 | 1.21 |

**Supplementary Table S4:** The standard method difference (SMD) between ESM and QAMS (%, n=3)

| No. | | Eriocitrin | | .Neoeriocitrin | | Narirutin | | Hesperidin | | Neohesperidin | | Hesperidin-7-O-glucoside | | Poncirin | | Naringenin | | Hesperetin | |
| --- | --- | --- | --- | --- | --- | --- | --- | --- | --- | --- | --- | --- | --- | --- | --- | --- | --- | --- | --- |
|  |  | ESM | QAMS | ESM | QAMS | ESM | QAMS | ESM | QAMS | ESM | QAMS | ESM | QAMS | ESM | QAMS | ESM | QAMS | ESM | QAMS |
| F1 | SMD | 1.78 | | -0.60 | | 0.13 | | 0.92 | | 1.99 | | 0.87 | | 1.50 | | 0.21 | | 0.99 | |
| F2 | SMD | 0.60 | | -1.56 | | -0.31 | | 1.68 | | 1.98 | | 0.72 | | 0.26 | | 1.77 | | 0.85 | |
| F3 | SMD | 0.26 | | -1.14 | | -0.24 | | 0.62 | | 0.23 | | 0.10 | | 1.30 | | -1.46 | | 1.15 | |
| F4 | SMD | -0.77 | | 1.38 | | -0.28 | | 0.38 | | 1.99 | | 1.27 | | -0.41 | | -1.50 | | 1.04 | |
| F5 | SMD | -0.75 | | -1.24 | | -0.44 | | 0.80 | | -0.64 | | 0.90 | | -1.17 | | -1.56 | | 0.51 | |
| S1 | SMD | 1.55 | | 0.59 | | 0.15 | | -0.10 | | 1.51 | | 1.28 | | 0.55 | | 1.30 | | 0.33 | |
| S2 | SMD | 1.30 | | 0.56 | | 0.91 | | -0.46 | | 1.33 | | 1.34 | | -0.08 | | 1.68 | | 1.26 | |
| S3 | SMD | 0.52 | | -0.06 | | -0.43 | | -1.59 | | 0.75 | | 0.35 | | -0.54 | | 1.32 | | 0.36 | |
| S4 | SMD | -0.73 | | -2.00 | | -0.20 | | -0.48 | | -0.73 | | 0.68 | | -0.30 | | 1.45 | | 0.52 | |
| S5 | SMD | 1.11 | | 0.18 | | -0.13 | | -1.41 | | 1.63 | | -0.03 | | 0.35 | | 1.28 | | 0.54 | |
| T1 | SMD | 0.86 | | 1.19 | | 0.65 | | -0.38 | | 1.71 | | 0.85 | | 1.06 | | -1.23 | | 1.09 | |
| T2 | SMD | 0.74 | | 1.50 | | 0.21 | | 1.31 | | 0.87 | | 0.56 | | -0.24 | | 1.01 | | 1.17 | |
| T3 | SMD | 0.60 | | 0.46 | | -0.31 | | 1.88 | | 1.18 | | 0.72 | | -0.23 | | 1.08 | | -0.15 | |
| T4 | SMD | -0.53 | | -0.49 | | -1.18 | | 0.13 | | 0.55 | | -0.06 | | -0.19 | | 0.96 | | 0.36 | |
| T5 | SMD | -0.23 | | -1.23 | | -0.59 | | 1.44 | | 0.64 | | -0.44 | | -0.11 | | 0.60 | | 0.41 | |
| H1 | SMD | 1.44 | | 1.50 | | 1.50 | | 1.26 | | 1.34 | | -0.04 | | 0.02 | | 1.06 | | -0.39 | |
| H2 | SMD | 1.36 | | 1.17 | | 1.19 | | 1.17 | | 1.36 | | 1.51 | | 0.02 | | 1.06 | | 0.10 | |
| H3 | SMD | 1.08 | | 0.54 | | 0.72 | | 0.04 | | 1.06 | | 1.42 | | -0.01 | | 1.13 | | 1.64 | |
| H4 | SMD | 0.78 | | 0.21 | | 0.34 | | -0.50 | | 0.89 | | 0.66 | | -0.53 | | 1.64 | | -1.14 | |
| H5 | SMD | 0.23 | | 0.17 | | 0.05 | | -1.09 | | 0.83 | | 1.77 | | -0.96 | | 1.80 | | 0.36 | |

**Supplementary Table S5** Comparison of the contents of ten flavonoids in samples from different fermentation time

| Fermentation time / d | Eriocitrin | Neoeriocitrin | Narirutin | Naringin | Hesperidin | Neohesperidin | Hesperidin-7-O-glucoside | Poncirin | Naringenin | Hesperetin |
| --- | --- | --- | --- | --- | --- | --- | --- | --- | --- | --- |
| RFA | 4.29 | 1.07 | 4.56 | 43.37 | 2.70 | 46.53 | 0.17 | 4.73 | 0.02 | 0.00 |
| F1 | 4.11 | 2.61 | 3.76 | 28.42 | 2.17 | 46.06 | 0.16 | 3.43 | 0.17 | 0.06 |
| F2 | 3.14 | 1.64 | 3.45 | 20.38 | 3.05 | 38.60 | 9.79 | 2.50 | 0.75 | 0.52 |
| F3 | 2.94 | 0.64 | 3.50 | 17.03 | 1.89 | 27.49 | 14.17 | 1.60 | 1.37 | 1.21 |
| F4 | 2.46 | 0.88 | 3.47 | 14.94 | 1.74 | 37.49 | 5.26 | 2.17 | 1.26 | 1.23 |
| F5 | 2.46 | 0.71 | 3.37 | 10.25 | 2.60 | 24.21 | 4.38 | 1.45 | 1.15 | 1.03 |

**Supplementary Table S6** Comparison of the contents of ten flavonoids in samples from different soaking time

| Soaking time / h | Eriocitrin | Neoeriocitrin | Narirutin | Naringin | Hesperidin | Neohesperidin | Hesperidin-7-O-glucoside | Poncirin | Naringenin | Hesperetin |
| --- | --- | --- | --- | --- | --- | --- | --- | --- | --- | --- |
| RFA | 4.29 | 1.07 | 4.56 | 43.37 | 2.70 | 46.53 | 0.17 | 4.73 | 0.02 | 0.00 |
| S1 | 3.88 | 0.95 | 3.78 | 18.02 | 2.30 | 41.11 | 6.40 | 3.01 | 0.96 | 0.98 |
| S2 | 3.66 | 0.94 | 4.47 | 24.08 | 2.22 | 39.52 | 10.18 | 2.57 | 1.81 | 1.46 |
| S3 | 3.09 | 0.89 | 3.38 | 18.58 | 1.98 | 35.12 | 6.69 | 2.32 | 0.98 | 0.80 |
| S4 | 2.47 | 0.75 | 3.52 | 18.89 | 2.21 | 27.18 | 5.18 | 2.45 | 1.18 | 0.79 |
| S5 | 3.50 | 0.90 | 3.57 | 20.93 | 2.02 | 42.31 | 4.22 | 2.86 | 0.93 | 0.79 |

**Supplementary Table S7** Comparison of the contents of ten flavonoids in samples from different fermentation temperatures

| Fermentation temperatures / °C | Eriocitrin | Neoeriocitrin | Narirutin | Naringin | Hesperidin | Neohesperidin | Hesperidin-7-O-glucoside | Poncirin | Naringenin | Hesperetin |
| --- | --- | --- | --- | --- | --- | --- | --- | --- | --- | --- |
| RFA | 4.29 | 1.07 | 4.56 | 43.37 | 2.70 | 46.53 | 0.17 | 4.73 | 0.02 | 0.00 |
| T1 | 3.32 | 1.01 | 4.21 | 22.99 | 2.24 | 43.04 | 0.16 | 3.48 | 0.23 | 0.18 |
| T2 | 3.23 | 1.08 | 3.82 | 22.31 | 2.71 | 35.94 | 3.08 | 2.48 | 0.71 | 0.45 |
| T3 | 3.14 | 1.69 | 3.45 | 20.38 | 3.06 | 38.30 | 5.26 | 2.49 | 0.75 | 0.52 |
| T4 | 2.55 | 0.67 | 2.96 | 13.31 | 2.36 | 33.79 | 4.19 | 2.51 | 0.67 | 0.50 |
| T5 | 2.69 | 0.80 | 3.28 | 15.05 | 2.75 | 34.34 | 1.57 | 2.55 | 0.51 | 0.40 |

**Supplementary Table S8** Comparison of the contents of ten flavonoids in samples from different fermentation humidity

| Fermentation humidity / % | Eriocitrin | Neoeriocitrin | Narirutin | Naringin | Hesperidin | Neohesperidin | Hesperidin-7-O-glucoside | Poncirin | Naringenin | Hesperetin |
| --- | --- | --- | --- | --- | --- | --- | --- | --- | --- | --- |
| RFA | 4.29 | 1.07 | 4.56 | 43.37 | 2.70 | 46.53 | 0.17 | 4.73 | 0.02 | 0.00 |
| H1 | 3.78 | 1.04 | 5.19 | 21.29 | 3.18 | 39.62 | 1.49 | 2.63 | 0.74 | 0.53 |
| H2 | 3.71 | 1.00 | 4.79 | 20.43 | 3.14 | 39.79 | 1.73 | 2.64 | 0.74 | 0.68 |
| H3 | 3.48 | 0.94 | 4.28 | 20.43 | 2.70 | 37.35 | 2.82 | 2.62 | 0.79 | 0.85 |
| H4 | 3.26 | 0.91 | 3.93 | 19.23 | 2.53 | 36.09 | 5.15 | 2.33 | 1.67 | 1.19 |
| H5 | 2.93 | 0.91 | 3.70 | 15.10 | 2.36 | 35.64 | 7.88 | 2.14 | 2.50 | 2.88 |
